# Long-Term Prognosis in Relation to Vitamin D Status in Pediatric Solid Tumor Patients

**DOI:** 10.3390/nu15214571

**Published:** 2023-10-27

**Authors:** Nóra Kárász, Orsolya Juhász, Marcell Imrei, Miklós Garami

**Affiliations:** 1 Faculty of Medicine, Semmelweis University, 1085 Budapest, Hungary; karasz.nora@stud.semmelweis.hu; 2 Pediatric Center, Semmelweis University, 1094 Budapest, Hungary; juhasz.orsolya@med.semmelweis-univ.hu; 3 Centre for Translational Medicine, Semmelweis University, 1085 Budapest, Hungary; marcell.imrei@gmail.com; 4 Heim Pál National Pediatric Institute, 1089 Budapest, Hungary

**Keywords:** Vitamin D, pediatric oncology, follow-up, relapse-free survival, overall survival, prognosis

## Abstract

Background: Hypovitaminosis D is associated with oncogenesis, and the initial level of Vitamin D may play a role in determining long-term prognosis, relapse-free survival (RFS) and overall survival (OS). The purpose of our study was to follow up pediatric cancer patients for a long time in terms of their baseline Vitamin D level and disease outcomes. Methods: We collected data on the initial 25(OH)D concentration in 117 children and examined their RFS and OS using Kaplan–Meier curves. Results: The initial 25(OH)D mean value in the relapsed group was 20.35 ng/mL (SE: 2.05) and in children without relapse it was 26.14 ng/mL (SE: 1.13). Both the relapse-free and overall Kaplan–Meier curves showed a tendency for children with lower serum Vitamin D concentrations to experience cancer recurrence or fatal outcomes sooner than patients with normal serum levels. Conclusions: Our results indicated a possible correlation between higher pretreatment serum Vitamin D concentrations and improved overall and relapse-free survival.

## 1. Introduction

Recently, uncountable data have shown that Vitamin D is not limited to the regulation of calcium, phosphate and bone homeostasis. Although at first glance, Vitamin D deficiency (VDD) is associated with rickets and osteomalacia, it is not only osteoblasts that express Vitamin D receptor, but also other cells of the body, such as β islet cells, cardiomyocytes, intestinal cells, lymphocytes, neurons, etc. [1]. This may explain the various extraskeletal effects and increased incidence of exacerbations of type 1 diabetes mellitus, inflammatory bowel disease, multiple sclerosis, respiratory infections and asthma in patients with low serum Vitamin D concentrations [2].

As numerous diseases are associated with Vitamin D deficiency, these pleiotropic effects raise the question of whether there is a link between Vitamin D status and cancer. This hypothesis was proposed even before the breakthrough in molecular biology, when scientists at Johns Hopkins University in the 20th century observed that the mortality rate of colon carcinoma in the northern part of the USA was higher than that in the southern part of the country, due to differences in latitude in ultraviolet B radiations and therefore the differences in Vitamin D levels [3]. Subsequently, an increasing number of studies have been published supporting the hypothesis that cancer and low concentrations of Vitamin D might be linked [4,5,6].

The active form of Vitamin D is 1,25-dihydroxy-cholecalciferol (1,25(OH)_2_D or calcitriol), which is a steroid hormone. Even though 1,25(OH)_2_D is the active hormone, laboratories usually measure the amount of 25-hydroxy Vitamin D (25(OH)D) to check Vitamin D levels. It is the predominant form of Vitamin D in the circulation, and one of the most accurate biomarkers of Vitamin D status [7,8]. The 25(OH)D serum concentration is referred to as Vitamin D level.

Most studies have examined the adult population, and information is limited on pediatric patients with malignancies. However, VDD is a huge problem among minors; therefore, it raises the issue of the manifestation of skeletal and extraskeletal abnormalities in their bodies, too, including malignant diseases [9]. With a focus on pediatric oncology, attempts have been made to create a systematic review of the available data on Vitamin D deficiency and insufficiency in children with cancer worldwide; however, as there are not enough standardized studies on the topic, it is difficult to obtain a global perspective [10].

Moreover, long-term follow-up studies are necessary to monitor the Vitamin D status of children months or years after the end of their treatment. It is important to note that an association between low Vitamin D levels and worse outcomes has been reported in the adult population. In both oncological and hematological diseases, better survival has been proven in patients with higher serum Vitamin D levels [11,12]. Furthermore, published data have highlighted a possible link between disease relapse and reduced calcitriol level. Researchers from the USA have observed an inverse correlation between Vitamin D consumption and the recurrence of head and neck cancer, which suggests a possible role of the hormone in determining long-term cancer outcomes [13].

Although a mounting number of studies indicate that adults with low concentrations of Vitamin D are at an increased risk for complications and adverse outcomes if they have a malignant disease, there is little information on children. The literature is limited, and existing data need to be supported by further studies [14].

We retrospectively used information from adult patients to conduct an exploratory study on childhood cancer outcomes. Our aim was to investigate the baseline Vitamin D status of children before treatment to determine whether there was a significant correlation between initial Vitamin D concentration and overall survival (OS). Moreover, the statistical relationship was examined between baseline circulating hormone levels and cancer recurrence and relapse-free survival (RFS). Our hypothesis predicted that pediatric cancer patients with higher hormone levels at diagnosis will have the highest OS- and RFS-rates.

## 2. Materials and Methods

### 2.1. Patients

Between 2007 and 2022, 117 children were recruited from among patients diagnosed, treated and followed-up for non-hematological malignancies at the Pediatric Center, Semmelweis University (SU). The examined population consisted of 58 male (49.5%) and 59 female (50.5%) children aged 0 to 18 (mean age: 5.03 years).

The underlying disease was heterogeneous. A total of 26 different tumor types occurred in the selected population, of which neuroblastoma (35 cases—29.9%), Ewing-sarcoma (18 cases—15.4%) and medulloblastoma (16 cases—13.7%) were overrepresented as the three most common malignancies in the patient group. Cancers beyond these three categories were underrepresented, with 8 nephroblastomas (6.8%), 5 retinoblastomas (4.3%), 4 hepatoblastomas (3.4%), 4 ganglioneuromas (3.4%), 3 teratomas (2.6%), 3 atypical teratoid rhabdoid tumors (ATRT) (2.6%), 2 primitive neuroectodermal tumors (PNET) (1.7%), 2 ependymomas (1.7%), 2 papillary thyroid carcinomas (1.7%), 2 astrocytomas (1.7%), 1 cerebral neuroblastoma (0.9%), 1 anaplastic astrocytoma (0.9%), 1 medullar thyroid carcinoma (0.9%), 1 testicular tumor (0.9%), 1 rhabdoid renal tumor (0.9%), 1 sinus ethmoidalis tumor (0.9%), 1 adrenal cortex carcinoma (0.9%), 1 tumor of the peripherial nerves and autonomous nervous system (0.9%), 1 glioneural tumor (0.9%), 1 optic glioma (0.9%), 1 choroid plexus carcinoma (0.9%), 1 plexus papilloma carcinoma (0.9%) and 1 pineoblastoma (0.9%). The distribution of pediatric tumor types in our patient population is shown in Table 1. The histopathological examination provided conclusive evidence for each diagnosis.

Of the 117 participants, 21 cases (17.95%) were found to have recurrence of the primary tumor. Seventeen cases were early recurrences, which means that recurrence occurred in the first two years after the completion of primary oncological treatment. In the remaining four cases, the tumor recurred after two years, resulting in late relapse. After secondary surgical or oncological treatment, 8 of the 21 children achieved complete remission (CR), 2 responded with partial remission (PR), 2 had stable disease (SD), and 9 had an endpoint of exitus letalis (EX). The remaining 96 children who had not relapsed remained in CR or had SD and were still alive at the end of the follow-up period or at the last follow-up appointment (Table 1).

Children in our patient population had been involved in a previous study conducted at the Pediatric Center, SU, in 2019. In that survey, the Vitamin D status of children with malignancies was compared to that of a healthy control group [15]. Patients who dropped out of the previous study and who did not attend regular annual check-ups after the completion of treatment were excluded from the original patient population. The exclusion criteria reduced the number of patients from 173 to 117.

As we had access to the National Childhood Cancer Registry of Hungary and the MedSolution system of SU, we used these websites as sources of the necessary data. Maintaining the complete anonymity of patients was a priority. Informed consent was obtained from all parents and participants. The ethical statement was approved by the Hungarian National Ethical Review Board.

Patients were followed up from the initial diagnosis of cancer, which is considered the start of survival. All children completed the primary oncological treatment and were followed up regularly. The median follow-up time of patients was 8.9 years. The end of the survival period was considered to be either the date of the contact with the individual or the date of death. All cancer-related deaths were included in this study.

At regular (monthly, bi- or tri-monthly, annual, etc.) follow-up appointments, physical examinations, laboratory tests and medical imaging tests were performed to detect changes in patient status. Medical imaging and occasional histological examinations confirmed the recurrence of the primary tumor. Children with recurrent disease were separated into two groups in the study: patients with and without relapse. In both groups, data of Vitamin D levels were collected.

### 2.2. Samples and Sample Collection

Non-fasting samples were collected from peripheral venous blood and serum 25(OH)D concentrations were measured. The first sample from each child used in this study was required after the diagnosis of childhood malignant disease but before the initiation of oncological treatment.

The initial blood samples and specimens were taken between 14 December 2007, and 16 October 2014, respectively.

### 2.3. Biochemical Analyses

Vitamin D levels were measured at the Department of Laboratory Medicine, SU. The serum concentrations of 25(OH)D were quantified using immunoanalytical methods and an electrochemiluminescence immunoassay (ECLIA). The Cobas 601 kit (Roche, Basel, Switzerland) and the Liaisonkit (DiaSorin, Saluggia, Italy) were used. Taking information from the literature and guidelines into account [10], we divided our patient population into two groups according to their initial serum 25(OH)D levels. If it was under 30 ng/mL, we considered it a low serum Vitamin D level, whereas if it was above 30 ng/mL we classified it as normal serum Vitamin D.

### 2.4. Statistical Analysis

For statistical analysis we used the statistical program ”R”, and we used ‘survival’, ‘ggplot2′ and ‘ggfortify’ libraries. Kaplan–Meier survival curves were created to demonstrate OS and RFS in both the low serum Vitamin D and normal serum Vitamin D groups. OS was defined as the period from diagnosis to death. The time between the primary treatment and the first recurrence of malignant disease is referred to as RFS. OS and RFS were plotted on Kaplan–Meier curves with 95% confidence intervals (CI). The starting point of the curves was the time of oncological treatment initiation. All censoring was marked with a vertical line. Initial Vitamin D levels were categorized into normal and low groups using a cut-off point uniformly used in the literature, and the resulting categorical variable was analyzed as an independent variable using Cox regression. The *p*-value obtained in the regression analysis was considered significant below 0.05.

## 3. Results

Of the examined patients, 49.5% (*n* = 58) were male and 50.5% (*n* = 59) were female children aged 0 to 18 (mean age: 5.03 years). In total, 94 children younger than 10 years and 23 adolescents were included.

As an indicator of nutritional status, we measured the body mass index (BMI) in 95 cases out of 117. The mean value of BMI was 16.3 kg/m^2^, while the mean BMI percentile was 43.2. A total of 14.74% of our patients suffered cachexia, as they were under the third percentile according to the Hungarian BMI percentile table.

**Figure 1:**
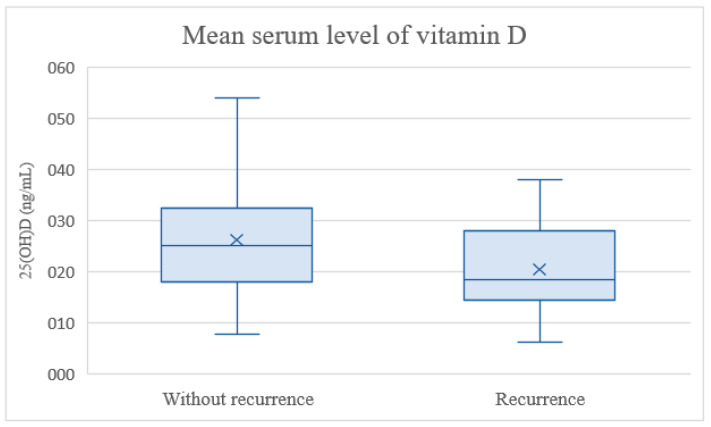
Baseline Vitamin D concentrations in children with relapsing disease and in patients in remission. The box extends from the first quartile to the third quartile. The horizontal line indicates the median, whereas mean values are marked with an X. The upper whisker goes to the maximum, lower whisker goes to the minimum.

The initial pretreatment Vitamin D value was 25.10 ng/mL (SE: 1.02). When serum Vitamin D levels were compared, the mean value in the group of patients without a relapse was 26.14 ng/mL (SE: 1.13), and in the relapse class it was 20.35 ng/mL (SE: 2.05) (Figure 1).

**Figure 2:**
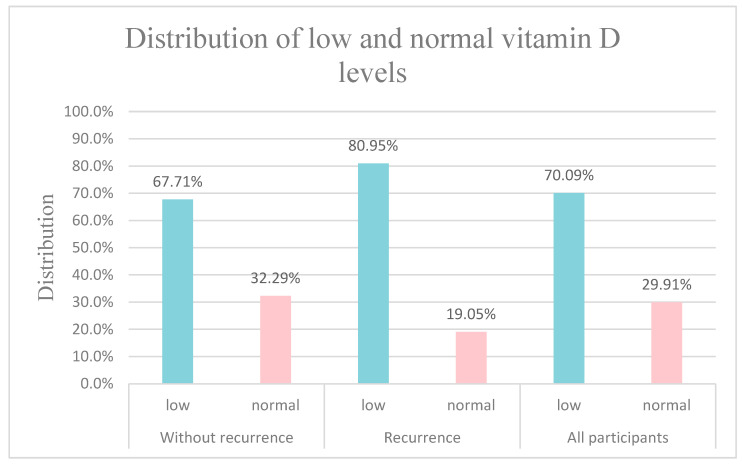
Whether children had recurrent malignant diseases or not, the prevalence of Vitamin D insufficiency was indeed high in our pediatric patient population. Children whose neoplasm had not relapsed tended to have higher Vitamin D concentrations.

According to international guidelines, the mean values in the whole patient population and separately in both groups were under 30 ng/mL, which is the lower limit of normal serum Vitamin D levels. Concentrations below this threshold are referred to as Vitamin D insufficiency [16]; in our study, we used the term low serum Vitamin D. In total, 82 (70.09%) of the 117 children had Vitamin D insufficiency, and 35 (29.91%) belonged to the normal serum Vitamin D group. Of the 96 children who did not have any recurrent disease, 65 of them (67.71%) belonged to the low serum Vitamin D group and 31 (32.29%) patients had serum Vitamin D levels above the threshold of 30 ng/mL. In the recurrence group, 17 (80.95%) were Vitamin D insufficient and only 4 (19.05%) children whose malignancy reappeared belonged to the normal serum Vitamin D class. The distribution of the Vitamin D status is summarized in Figure 2.

The studied population was very heterogeneous according to the histological diagnosis, and many types of malignancies were represented in only one or two cases. Therefore, only the three most common cancers in our patient population were examined separately in association with the Vitamin D levels. The highest Vitamin D level was found in a child with neuroblastoma: 69.50 ng/mL. The lowest concentration was 6.20 ng/mL, which was detected in a patient with Ewing’s sarcoma. No significant difference was observed among the three most common tumor types with respect to the mean Vitamin D levels. The mean concentrations were 25.89 ng/mL (SE: 1.81) among neuroblastoma patients, 24.74 ng/mL (SE: 2.73) among children with Ewing’s sarcoma and 22.20 ng/mL (SE: 2.71) among medulloblastoma patients.

Examining the Vitamin D status in smaller children and adolescents, we did not find a significant difference as the mean Vitamin D level was 22.76 ng/mL in children older than 10 years and 25.67 ng/mL in smaller children. A total of 82.61% of the adolescents and 67.02% of the younger patients had inadequate Vitamin D levels.

In total, 42% of the samples were taken in summer and spring, while the remainder were taken in autumn and winter. The highest concentrations were measured during the summer months as the mean level of Vitamin D was 28.86 ng/mL. Data on the lowest concentrations were collected during winter, with a 22.37 ng/mL mean level.

**Figure 3:**
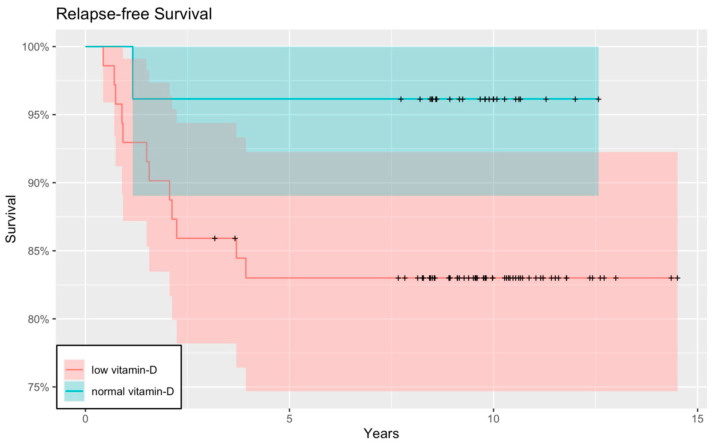
RFS with a Kaplan–Meier curve. When we compared the RFS between children with adequate and inadequate Vitamin D status, a worse RFS was detected in the latter group. The difference between the two groups shows a tendency, but the result is not significant (*p* = 0.137). The red and blue regions represent the 95% confidence intervals for survival rates in the low-vitamin-D group and the normal-vitamin-D group, respectively.

**Figure 4:**
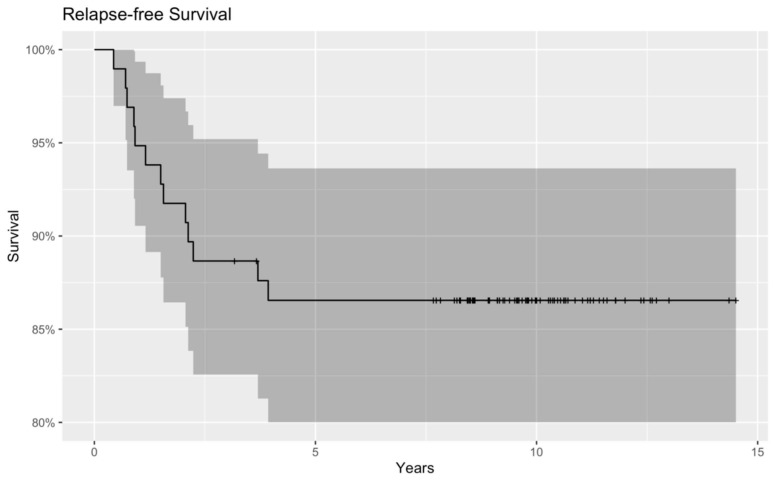
RFS of the whole patient population (normal and low serum Vitamin D groups together) with a Kaplan–Meier curve. Dark grey region represents the 95% confidence intervals for the whole patient population.

Moreover, as one of our main objectives, we constructed Kaplan–Meier curves to obtain and visualize the survival information. The relapse-free Kaplan–Meier curve showed that children with low serum Vitamin D levels tended to have cancer recurrence sooner, and therefore worse RFS than patients with normal serum Vitamin D. Although the curve shows a tendency, the results are not significant (*p* = 0.137) (Figure 3 and Figure 4).

**Figure 5:**
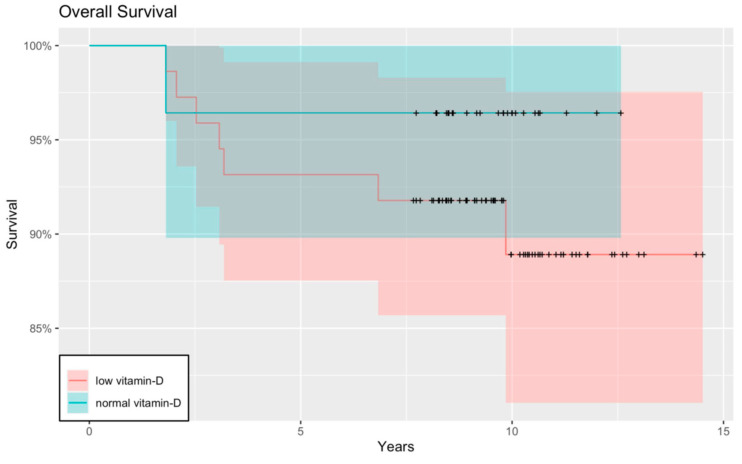
OS with a Kaplan–Meier curve. When we compared the OS between children with adequate and inadequate Vitamin D status, a worse OS was detected in the latter group. The difference between the two groups shows a tendency, but the result is not significant (*p* = 0.359). The red and blue regions represent the 95% confidence intervals for survival rates in the low-vitamin-D group and the normal-vitamin-D group, respectively.

**Figure 6:**
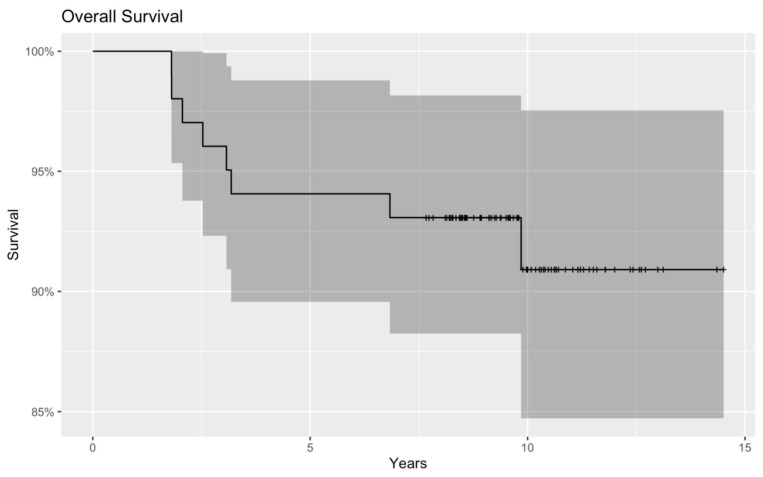
OS of the whole patient population (normal and low serum Vitamin D groups together) with a Kaplan–Meier curve. Dark grey region represents the 95% confidence intervals for the whole patient population.

The Kaplan–Meier analysis of OS showed a similar outcome: OS was likely to be greater with higher Vitamin D concentrations than with Vitamin D insufficiency. However, it did not meet the criteria for a significant correlation (*p* = 0.359) (Figure 5 and Figure 6).

## 4. Discussion

Approximately 1 billion people, or 14% of the population of the world, including children, adolescents and adults, do not have adequate levels of Vitamin D [17]. The risk of Vitamin D insufficiency and deficiency is significant in the general underage population [18], although it may be considerably higher in children with cancer [15,19]. Our study supports the hypothesis of a high prevalence of hypovitaminosis in pediatric cancer, as 70.09% of our study population had Vitamin D insufficiency at diagnosis. However, it is challenging to compare our findings with those of different studies because the majority of them used different patient groups at different stages, focusing on diagnostic, treatment or post-therapeutic processes. Genc et al. highlighted the problem of non-standardized patient populations. Similar to us, they also measured the initial, pre-treatment 25(OH)D concentrations and obtained similar results, with 63% of their underaged patients deficient in Vitamin D status [20]. Jackmann et al. conducted a similar study to ours, but the examined children had hematological malignancies, in contrast with our study, as our patient population suffered from solid tumors. According to their findings, one third of the patients had low baseline Vitamin D concentration, while in our study it was more than 70%. However, we must highlight that the threshold limits were different, with 25(OH)D levels <20 ng/mL and <30 ng/mL. In both studies, older age was associated with decreased Vitamin D concentration; in our adolescent patients, the prevalence of decreased Vitamin D level was 82.61% [14].

As the connection between Vitamin D level and incidence and mortality of cancer has been the center of interest for a long time, several large international studies have been conducted on the topic. Recently, a meta-analysis by Guo et al. showed an association between adequate Vitamin D supplementation and reduction in total cancer mortality, but not in total cancer incidence [21]. The results were consistent with a previous meta-analysis conducted in 2018. Vitamin D supplementation reduced total cancer mortality and extended overall survival [5].

The pre-diagnostic or pre-treatment significance of Vitamin D levels has been investigated and highlighted in adult cancer patients. Most studies confirmed that OS was better after years of follow-up if the baseline Vitamin D status was higher [22,23,24]; however, one exception questioned this hypothesis [25]. Borchmann et al. examined OS and RFS in patients with Hodgkin’s lymphoma (HL) and found a significant correlation between initial VDD and higher rates of tumor recurrence [26]. Although our results are not significant, there is a tendency for better OS and RFS with higher pre-treatment Vitamin D concentrations, which corresponds to the data found in the literature mentioned above. Nevertheless, in most cases, the study population consisted of people older than 18 years, and information about children was limited. To date, only one study has focused on the connection between pre-diagnostic Vitamin D levels in children with solid tumors and overall survival. The results are controversial and we must emphasize that researchers have measured 1,25(OH)_2_D levels [27] and not 25(OH)D, which is a widely accepted biomarker of Vitamin D status [7,8]. Consequently, to our knowledge, our study is the first to cover such a long period of time and to go back many years in patient histories to analyze the data and demonstrate both OS and RFS in correlation with baseline 25(OH)D concentrations.

Recent research has revealed that vitamins can modify the genome through several molecular pathways. They can alter the function of oncogenes and transcription factors by inhibiting cell proliferation, inducing apoptosis, suppressing growth factors, regulating autophagy and increasing cell differentiation [28]. The active hormone arrests the cell cycle in the G1/G0 phase, leading to the inhibition of cell proliferation by blocking entry into the S (DNA replication) phase [29]. Cell cycle arrest occurs via the genomic regulations of cyclins, cyclin-dependent kinases (CDKs) and CDK-inhibitors [30]. Moreover, BCL2 and BCL-XL suppress anti-apoptotic mediators and BAX, BAK and BAD are pro-apoptotic proteins activated in the presence of 1,25(OH)_2_D [31]. Angiogenesis is an important step in tumor expansion, invasion and metastasis [32]. However, Vitamin D downregulates the expression of HIF-1α, which plays a prominent role in the transcription of VEGF, a growth factor that regulates angiogenesis.

As immuno-oncology has become a promising field in the research of cancer, the functions of the immune system in the prevention and suppression of tumors have been investigated. The immune system eliminates emerging tumor cells, it combats viral infections and reduces the risk of tumors caused by viruses, and it eradicates pathogens and inhibits the inflammation that promotes tumor formation. Vitamin D plays a regulatory role in cells pertaining to both the innate and adaptive components of the immune system [33]. Vitamin D functions as a stimulator of the innate immune response, modulating the expression of genes encoding anti-microbial proteins such as cathelicidin [34]. However, the hormone also influences the adaptive immune system, especially the T-cell mediated immunological reactions. Vitamin D inhibits nuclear factor-kappa B (NF-κB) signaling, which is crucial for the activation of T-helper cells. NF-κB is a ubiquitous transcription factor that drives inflammation. It is blocked because Vitamin D enhances the expression of the inhibitor of nuclear factor kappa B (IκB), which is an inhibitory protein of the transcription factor [30]. Moreover, Dimitrov et al. showed that Vitamin D upregulates the genes of programmed death-ligand 1 and 2 (PD-L1 and PD-L2) in epitheloid and myeloid cells [35]. The significant relevance of this finding is in the use of combination therapy of Vitamin D and immune checkpoint inhibitors (ICIs). The more PD-L1 is expressed, the more efficient the ICI therapy is [36].

When Vitamin D receptor (VDR)-knockout mice were examined in in vivo experiments, increased cell proliferation was observed. As active Vitamin D cannot initiate an antiproliferative signaling pathway due to the lack of VDR expression, the mitosis rate increases, and premalignant and malignant transformations are induced [37,38]. However, both the presence and quantity of the expressed receptors are crucial. According to several studies, the amount of VDR present might be predictive of malignant disease outcomes, as studies have shown that the higher the VDR expression in tumor tissue, the better the prognosis of patients. A possible reason for this phenomenon is that VDR becomes more sensitive to downregulation as the tumor progresses into a more advanced stage [39,40,41,42].

Our research aimed to learn more about the correlation between Vitamin D levels and cancer itself, rather than the effects of therapy on Vitamin D status. On the other hand, we cannot ignore the fact that after diagnosis, when oncological therapy has been introduced, physicians must expect and be alert to a significant reduction in Vitamin D level, because the adverse effects of cytotoxic drugs reduce serum Vitamin D concentrations in many ways. Hospitalization and phototoxic chemotherapy might limit the time spent by children outdoors, reducing their exposure to the sun [43]. Even if they can spend time outdoors, they must use sunscreen, which attenuates endogenous hormone synthesis [44]. Reduced physical activity and altered eating habits are common as a result of both malignancy and oncological treatment [45], and chemotherapy-induced inflammation of the intestinal mucosa may result in Vitamin D not being absorbed from the diet [46]. Glucocorticoids are an important part of therapy, to combat hematological diseases [47] and solid tumors [48]. However, in addition to the well-known symptoms of iatrogenic Cushing’s syndrome, glucocorticoids are responsible for suppressing the effects of calcitriol, resulting in a VDD-like condition [49]. Another unintended consequence of chemotherapeutic treatment is impaired Vitamin D synthesis in the liver and kidneys due to damage in the hepatic and renal tissues [10]. Nevertheless, after oncological treatment has been completed, we would expect Vitamin D status to improve as the body recovers from the disease and is no longer exposed to cell-destroying agents. The literature is limited on the Vitamin D status of childhood cancer survivors. In survivors from a southern Thai population, Vitamin D levels were low in comparison to those of healthy Europeans and North Americans. The authors noted that the lower levels of their population might partly reflect geographic differences in Vitamin D metabolism [50]. A study systematically followed childhood cancer survivors and observed a steady decline in Vitamin D levels over the survival period. Surprisingly, most individuals who were found to have normal baseline Vitamin D levels still had hypovitaminosis D by the end of the follow-up period. The explanation for this finding is not fully understood, but residual organ lesions and compliance problems with Vitamin D supplementation in patients might be possible causes [51].

One of the biggest concerns for cancer survivors is the recurrence of malignant disease. A remarkable finding is that there is a psychological phenomenon called fear of cancer recurrence (FCR), which has also been observed in pediatric patients, too [52]. Five years of disease-free survival is declared cured, which means the patient’s risk of dying from their neoplasm is statistically equivalent to the risk of dying from any other cause in the normal population [53]. However, according to the Childhood Cancer Survivor Study (CCSS), the most common cause of death for those who remained cancer-free beyond the first five years after the treatment was completed was a recurrence of the primary malignancy, even up to 20 years after the initial diagnosis [54]. Brain tumor and Ewing’s sarcoma recurrence were reported to have the highest incidence, whereas patients with renal malignancies (Wilms tumor) had the lowest risk [55].

Given these problems, the early detection of recurrent disease, cancer lesions and treatment-related morbidity is of cardinal importance. As the survival rate of pediatric cancer is increasing, the demand for organized long-term follow-up care is essential. The aim of survival care is to offer childhood cancer survivors access to regular follow-up meetings with physicians, treatment for possible late effects of chemo- and radiotherapy, education and psychological help to empower them. Providing individualized surveillance should be the optimum [56]. The long-term surveillance of children who have overcome cancer begins with regular (monthly, bi-, tri-monthly, annual, etc.) follow-up appointments, where physical examinations, laboratory tests, medical imaging and other necessary examinations are performed to detect any abnormalities. Survival time is a complex period, and its implementation is not as simple as expected. Firstly, it is a team effort involving pediatric oncologists, primary care physicians and other specialists such as cardiologists, nephrologists and neurologists, and it requires continuous consultations between them. Moreover, as children reach a certain age, pediatricians are no longer available to provide survival follow-ups; therefore they need to help their patients transition to the adult health care system [57]. Another problematic aspect of surveillance is the length of the survivorship period, as it is not clear how long such monitoring should last [55]. Tumor type, grade of malignancy, treatments, other diseases, and the compliance of patients and parents are all determining factors that may affect the follow-up time. Despite all the questions and difficulties associated with appropriate follow-up protocols, we can claim that regular follow-ups of patients are required for years.

### 4.1. Strengths and Limitations

The greatest strength of the study is the exceptionally long follow-up time. In view of the paucity of the literature in this patient population, the analysis conducted on the data of the pediatric population also distinguishes this study as exceptional.

A limitation of this paper is a lack of information on the ethnic origins of the patients, particularly those of Romani origin. Vitamin D levels are naturally lower in darker-skinned populations, who have adapted to lower levels by using Vitamin D more sparingly and more efficiently. This is notably the case with South Asian populations, from whom the Romani are derived [58]. Since the Romani have poorer health outcomes due to a variety of socioeconomic factors, our findings may be an artefact of such factors.

Since Vitamin D levels were not measured at the time of tumor recurrence, we were unable to include these data in our analysis. Another limitation of the study is that although our results disclosed a spectacular trend, they were not statistically significant; therefore, additional research is required to draw broad conclusions.

### 4.2. Implications on Practice and Research

As Vitamin D status is an important modifiable risk factor that can be targeted for prevention in healthy individuals and for a more favorable prognosis and overall and relapse-free survival in pediatric cancer, its normalization and Vitamin D supplementation are widely recommended. Low Vitamin D concentration is not only a possible factor in oncogenesis, but might also affect tumor progression, cancer recurrence, and disease outcome. Therefore, it is advisable to monitor the serum Vitamin D levels at the time of diagnosis.

As data on childhood cancer survival are limited in terms of the pre-diagnostic Vitamin D level of patients, further investigation is required on the topic, with larger sample sizes and even throughout therapy and later, during the survival period. Moreover, the authors would like to underline the importance of identifying the ethnic origins of future patients, particularly those from darker-skinned or Arctic populations that have adapted to lower levels of Vitamin D over tens of thousands of years [58].

## 5. Conclusions

Our investigation examined the problem of Vitamin D insufficiency and deficiency in children with malignant diseases and focused on the potential effect of the initial Vitamin D status on long-term outcomes. We were able to occasionally follow up pediatric cancer survivors beyond 10 years and detect progressive or recurrent neoplasms. Consistent with the current literature, our results indicate a possible correlation between higher pre-treatment serum Vitamin D concentrations and improved overall and relapse-free survival, suggesting that the initial serum Vitamin D level is a potential predictor of disease-free and relapse-free survival in pediatric cancer patients.

## Figures and Tables

**Table 1:** Outcomes of malignant diseases in the study population, separated by children who had a recurrence and those who did not. Outcomes of malignancies according to the histopathological diagnosis. ATRT = atypical teratoid rhabdoid tumor; PNET = primitive neuroectodermal tumor.

	Number of Patients	CR	PR	SD	EX
Relapsed *	21	8	2	2	9
Not relapsed	96	90	-	6	-
Neuroblastoma	35	30	-	3	2
Ewing sarcoma	18	16	-	-	2
Medulloblastoma	16	14	-	-	2
Nephroblastoma	8	8	-	-	-
Retinoblastoma	5	3	-	2	-
Hepatoblastoma	4	4	-	-	-
Ganglioneuroma	4	4	-	-	-
Teratoma	3	3	-	-	-
ATRT	3	1	-	1	1
PNET	2	2	-	-	-
Ependymoma	2	2	-	-	-
Papillary thyroid Carcinoma	2	1	1	-	-
Astrocytoma	2	2	-	-	-
Cerebral neuroblastoma	1	1	-	-	-
Anaplastic astocytoma	1	1	-	-	-
Medullar thyroid carcinoma	1	1	-	-	-
Testicular tumor	1	1	-	-	-
Rhabdoid renal tumor	1	1	-	-	-
Ethmoid sinus tumor	1	1	-	-	-
Adrenal cortex carcinoma	1	1	-	-	-
Tumor of the peripheral nerves and autonomous nervous system	1	1	-	-	-
Glioneural tumor	1	-	-	1	-
Optic glioma	1	-	-	1	-
Choroid plexus carcinoma	1	-	-	-	1
Plexus papilloma carcinoma	1	-	-	-	1
Pineoblastoma	1	-	1	-	-

* Secondary outcomes were investigated in patients who relapsed. The secondary outcomes were the outcomes of recurrent diseases. CR = complete remission; PR = partial remission; SD = stable disease; EX = exitus letalis.

## Data Availability

Not applicable.

## Abbreviations (in order of appearance):

RFS	relapse-free survival
OS	overall survival
SE	standard error
VDD	Vitamin D deficiency
1,25(OH)_2_D	1,25-dihydroxy-cholecalciferol
25(OH)D	25-hydroxy Vitamin D
SU	Semmelweis University
BMI	body mass index
ATRT	atypical teratoid rhabdoid tumor
PNET	primitive neuroectodermal tumor
CR	complete remission
PR	partial remission
SD	stable disease
EX	exitus letalis
ECLIA	electrochemiluminescence immunoassay
HL	Hodgkin’s lymphoma
CDK	cyclin-dependent kinase
NF-κB	nuclear factor-kappa B
IκB	inhibitor of nuclear factor kappa B
PD-L1	programmed death-ligand 1
ICIs	immune checkpoint inhibitors
VDR	Vitamin D receptor
FCR	fear of cancer recurrence
CCSS	Childhood Cancer Survivor Study
